# Methyl Ferulic Acid Attenuates Human Cardiac Fibroblasts Differentiation and Myocardial Fibrosis by Suppressing pRB-E2F1/CCNE2 and RhoA/ROCK2 Pathway

**DOI:** 10.3389/fphar.2021.714390

**Published:** 2021-08-17

**Authors:** Rongheng Liao, Zhen Qi, Ri Tang, Renrong Wang, Yongyi Wang

**Affiliations:** ^1^ Department of Cardiovascular Surgery, School of Medicine, Renji Hospital, Shanghai Jiao Tong University, Shanghai, China; ^2^ Department of Critical Care Medicine, School of Medicine, Renji Hospital, Shanghai Jiao Tong University, Shanghai, China; ^3^ Department of Cardiology, Wuxi No. 2 Hospital, Nanjing Medical University, Wuxi, China

**Keywords:** myocardial infarction, methyl ferulic acid, cardiac fibrosis, TGF-β1, E2F1

## Abstract

**Background:** Myocardial fibrosis is a key pathological process after myocardial infarction, which leads to poor outcomes in patients at the end stage. Effective treatments for improving prognosis of myocardial fibrosis are needed to be further developed. Methyl ferulic acid (MFA), a biologically active monomer extracted and purified from the Chinese herbal medicine, is reported as an attenuator in many diseases. In this study, we aim to reveal the role it plays in myocardial fibrosis after myocardial infarction and its possible mechanism.

**Results:** Firstly, we found that MFA attenuated the expression of fibrosis-related proteins and the ability of migration and proliferation in TGF-β1–induced human cardiac fibroblasts (HCFs). Then, myocardial fibrosis after myocardial infarction models on mouse was built to reveal the *in vivo* affection of MFA. After 28 days of treatments, fibrosis areas, cardiac function, and expression of fibrosis-related proteins were all improved in the MFA-treated group than the myocardial infarction group. Finally, to elucidate the mechanism of phenomenon we observed, we found that MFA attenuated HCF differentiation after myocardial infarction by suppressing the migration and proliferation in HCFs, which was by suppressing the pRB-E2F1/CCNE2 and the RhoA/ROCK2 pathway.

**Conclusion:** Our findings showed that MFA attenuated the expression of fibrosis-related proteins, and the ability of migration and proliferation in HCFs improved the cardiac function of myocardial infarction mice; meanwhile, the mechanism of that was by suppressing the pRB-E2F1/CCNE2 and the RhoA/ROCK2 pathway.

## Introduction

Myocardial infarction (MI) remains to be the leading cause of mortality in both developed countries and developing countries like China, especially in acute myocardial infarction (AMI) cases (Xu et al., 2020). After MI, myocardial ischemia of vasculature destruction leads to extensive cell death and apoptosis in affected myocardial and thus initiate myocardium remodeling. To revitalize the damaged issue, revascularization treatments have been considered as an effectual approach to avoid cardiac remodeling and improve cardiac function (Gao et al., 2019). Despite that the mortality of AMI patients has now decreased in some countries due to development of an emergency medical service, rational medication usage, and worldwide adoption of early reperfusion therapy (Yeh et al., 2010; Laribi et al., 2014), the myocardial fibrosis following MI is still associating with poor prognosis and bad outcome in patients at the end stage of MI (van den Borne et al., 2010).

Myocardial fibrosis is characterized by excessive deposition of the extracellular matrix (ECM) in the infarcted cardiac area. Secreted by cardiac fibroblasts, ECM plays a vital role in tissue reconstruction of the cardiac area in order to avoid heart rupture, but excessive ECM deposition may end up with reduced ventricular compliance and stiffened tissue, hence affecting cardiac function and causing heart failure at the end stage (Ma et al., 2018; Fan et al., 2019). α-smooth muscle actin (α-SMA), collagen type I (Col-1a1, approximately 85%), collagen type III (Col-III, approximately 11%), and fibronectin-1 (FN1) are the major characterized components of ECM (Kawaguchi et al., 2011; Travers et al., 2016; Hinderer and Schenke-Layland, 2019); expression of those proteins reflects the level of ECM secretion. Differentiation of cardiac fibroblasts plays an important role in myocardial fibrosis; stimulated by variety of cytokines, cardiac fibroblasts differentiate into myofibroblasts (Ma et al., 2018), which is more active in secreting ECM and other fibrosis-associated proteins. Transforming growth factor-β1 (TGF-β1) is considered as one of the most effective cytokines in this process, and TGF-β1 and its receptor TGFβRs involves together in myocardial fibrosis is widely reported (Bujak and Frangogiannis, 2007; Dobaczewski et al., 2011; Ma et al., 2018). Despite the protein expression of ECM, increasing abilities of cell proliferation and migration are also considered as key characters in differentiation of cardiac fibroblasts (Manabe et al., 2002). In recent years, to decrease myocardial fibrosis, some strategies have been developed to target TGF-β pathways, such as TGF-β inhibitors and anti-TGF-β antibodies (Cunnington et al., 2009; Lei et al., 2011), but they all failed to prevent myocardial fibrosis due to a failure in preventing TGFβ from binding to TGFβRs. Besides, the clinical use of these inhibitors for anti-fibrotic therapy was limited because of high toxicity (Akhurst and Hata, 2012). Hence, searching for safe and stable intervention targets for preventing and treating with human cardiac fibrosis is necessary.

Methyl ferulic acid (MFA), an organic acid extracted from traditional Chinese herbs like *Securidaca inappendiculata hasskarl*, is often served as anti-inflammatory, antibacterial, and anti-rheumatism remedies in reports (Cheng et al., 2019a). In recent years, the effect of MFA in ameliorating hepatic insulin resistance (Cheng et al., 2019b), protecting liver oxidative injury (Li et al., 2017), and reducing cell apoptosis (Li et al., 2018) were observed in some research groups, but its effects on attenuating myocardial fibrosis and its possible mechanism were not revealed yet. In our research, we aimed to determine the effect of MFA in attenuating myocardial fibrosis and reveal its possible mechanism by means of TGF-β1–induced human cardiac fibroblasts (HCFs) *in vitro* and myocardial fibrosis mice *in vivo*. Our data showed that MFA attenuated the expression of fibrosis-related proteins, suppressed the ability of migration and proliferation in HCFs, and improved the infarction sizes, cardiac function, and fibrosis-related protein expressions in myocardial fibrosis mice. The mechanism of that was by suppressing proliferation and migration ability of cardiac fibroblasts, which was by suppressing the pRB-E2F1/CCNE2 and the RhoA/ROCK2 pathway.

## Materials and Methods

### Reagents and Chemicals

Methyl ferulic acid (MFA) was obtained from the Sigma Chemical Co. (St. Louis, MO, United States), after purchasing from the agent, MFA was dissolved in dimethyl sulfoxide (DMSO) (Xilong Chemical Co., Ltd., Guangdong, China) for cell procedure and in DMSO + PEG300 (Xilong Chemical Co., Ltd., Guangdong, China) + ddH2O for mouse injection. TGF-β1 was obtained from Sino biology CO. (Beijing, China). Anti–FN-1 (Rabbit, 1:3,000, Abcam, MA, United States), anti–Col-1a1 (Rabbit, 1:1,000, Abclonal, Wuhan, China), anti–α-SMA (Rabbit, 1:1,000, Abcam, MA, United States), anti-pRB (Rabbit, 1:1,000, Abclonal, Wuhan, China),anti-E2F1 (Rabbit, 1:1,000, Abclonal, Wuhan, China), anti-CCNE2 (Rabbit, 1:1,000, Abclonal, Wuhan, China), anti-ROCK2 (Rabbit, 1:1,000, Abclonal, Wuhan, China), anti–p-MYPT1 (Rabbit, 1:1,000, Abclonal, Wuhan, China), anti-RhoA (Mouse, 1:100, Santa-Cruz, United States), and anti–α-tubulin (Rabbit, 1:3,000, CST, MA, United States) antibodies were obtained for Western blot and immunohistochemistry. Anti-FN-1 (Rabbit, 1:100, Abcam, MA, United States) and anti-α-SMA (Mouse, 1:100, Abcam, MA, United States) antibodies were obtained for immunofluorescence. Anti-pRB (Rabbit, 1:100, Protein-Tech, Wuhan, China) antibody was obtained for immunoprecipitation. The goat anti-rabbit IgG and goat anti-mouse IgG secondary antibodies were obtained from the Biotech Co., Ltd. (Beijing, China).

### Cell Culture and Transfection

Human cardiac fibroblasts (HCFs) were purchased from Sciencell Research Lab (6300, United States), HCFs were cultured in DMEM/F-12 medium with 5% fetal bovine serum (FBS, Gibco, United States), and 1% penicillin/streptomycin (Gibco, United States). After four days of culturing on condition of 37°C with 5% CO2, HCFs were incubated in MFA and TGF-β1 after 6 h of serum-free treatment, the concentrations of MFA and TGF-β1 were given in RESULTS. Before transfecting, E2F1-pcDNA 3.1 (100 nM), RhoA-pcDNA3.1 (100 nM), and NC-pcDNA3.1 (100nM, Sangon, Shanghai, China) were incubated respectively in lipofection 3000 (Sangon, Shanghai, China) under the room temperature, and after 20 min of incubating, the mixes were infected transiently into HCFs. After 48 h of transfecting, the HCFs were harvested for next procedure.

### Western Blot Analysis

HCFs were collected after treatments, cell lysates were harvested by RIPA (Beyotime, China), and the concentrations of cell lysates were determined by the BCA protein assay kit (Beyotime, United States). Same amounts of protein samples were separated by 10% SDS-PAGE gels and then transferred into the PVDF membrane. After blocking 1 h with 5% bovine serum albumin (BSA, Thermofisher, United States), the membranes were incubated separately in primary antibodies in condition of 4°C overnight. After washing three times in TBST for 15 min, the membranes were incubated in horseradish peroxidase (HRP)–conjugated secondary antibody (Goat to rabbit/mouse, Beyotime, China) for 2 h under the temperature of room. After washing three times in TBST for 15 min, membranes were imaged by enhanced chemiluminescence (ECL, Vazyme, Nanjing, China) and image data were analyzed by ImageJ software (NIH, MD, United States).

### Quantitative Realtime PCR Analysis

HCFs were collected after treatments, and total mRNAs were extracted by the EZ-press RNA Purification Kit (EZ-bioscience, United States) guided by operating instruction. After determining the concentrations, the mRNAs were reverse transcribed by the HiScript II RT SuperMix for qPCR (+ gDNA wiper) cDNA Synthesis Kit (Vazyme, Nanjing, China). Same amounts of total cDNAs were reacted in a 384-well qPCR plate with ChamQ SYBR GREEN qPCR Master Mix (Vazyme, Nanjing, China). The sequencing of primers in our research was given in Supplementary Table 1.

### Cell Viability Assay (CCK-8 Assay)

HCFs were cultured in 96-well plates. After treatments, the Cell Counter Kit-8 (CCK-8) assay (Vazyme, Nanjing, China) was used for evaluating cell viability guided by operating instruction. HCFs were incubated for 2 h after adding 1 μl CCK-8 solution into each well. A DNM-9602 enzyme-labeled analyzer (Beijing Perlong New Technology Co., Ltd.) was used for measuring optical density (OD) of each well. Six wells in each group were measured as replicates for the calculation of average cell viability.

### Cell Proliferation Assay (EDU Assay)

HCFs were cultured in 12-well plates. After treatments, the EDU Alexa Flour 564 imaging kit (Ribobio, Guangzhou, China) was used for detecting the proliferation of HCFs guided by an operating instruction. HCFs were incubated for 2 h after adding 1 μl EDU solution into each well. After incubating, HCFs were fixed in 75% ethyl alcohol on condition of −20°C overnight, then, fixed cells were penetrated by 0.3% PBST for 30 min, after washing three times in 0.1% TBST for 15 min, HCFs were incubated in EDU click solution (with Alexa Flour 564) away from light for 30 min. The images were captured by a fluorescence microscope (magnification: ×400; Olympus, Japan). The proliferation ability of HCFs was calculated by counting the percentage of EDU positive cells (red fluorescence) to DAPI stained cells (blue fluorescence).

### Cell Cycle Analysis (Flow Cytometry)

HCFs were collected after treatments and fixed in 75% ethyl alcohol on condition of −20°C overnight. After three times of washing and centrifuging (in 600 rpm, 5 min), fixed cells were stained by the Annexin V FITC/PI apoptosis and cell cycle kit (Multi Science, China) for 30 min, then, HCFs were analyzed by a flow cytometer (BD Biosciences, United States). Images were processed by FLOWJO software (BD Biosciences, United States), and the percentage of G0/G1, S, and G2/M were calculated to analyze cell cycle.

### Wound Healing Migration Assay

HCFs were cultured in 6-well plates. After culturing to 90% confluence, the plates were scratched by a 200 μl pipette tip led by a scratch ruler. After treating and culturing for another 24 h, the images of each same place were captured at 0 and 24 h by an inverted light microscope (magnification: ×100, Olympus, Japan), and the percentage of wound closure was accessed to evaluate the migration ability of HCFs.

### Transwell Migration Assay

HCFs were collected after treatments and then suspended with 500 μl DMEM/F-12 medium without serum. HCFs were placed equally into the upper chamber of Transwell plates (Corning, United States), and 2 ml of DMEM/F-12 with 20% serums were placed into the lower chamber. After 24 h of incubating, cells in lower side of membrane were fixed in 4% formaldehyde and penetrated by 0.3% PBST for 30 min; after washing three times in 0.1% TBST for 15 min, HCFs were stained with crystal violet (Beyotime, China), the images of each membrane were captured by inverted light microscope (magnification: ×100, Olympus, Japan), and the number of transferred cells was accessed to evaluate the migration ability of HCFs.

### Immunoprecipitation

HCFs were collected after treatments, concentrations of cell lysates were determined by the BCA protein assay kit, and 1 mg of proteins of each sample were used for immunoprecipitation. The samples were incubated in rabbit-controlled Ig-G antibody and anti-pRB (1:100) antibody under condition of 4°C overnight, and then the samples were suspended with 25 μl of Protein A/G PLUS-Agarose; after 2 h of incubating and washing, samples were Western blotted and anti-E2F1 (1:1,000) antibody was incubated to evaluate the conjunction amount of E2F1 and pRB.

### Experimental Animals and MI Modeling

C57BL/6 J wild type mice, which aged between 6–8 weeks, were purchased from the Institute of Laboratory Animal Science, Chinese Academy of Medical Sciences (Beijing, China). The mice were fed in SPF-graded animal house for 28 days with tap water and commercial diet available after MI modeling procedure. Mice were anesthetized with 3% isoflurane *via* inhalation and kept ventilated (Harvard Rodent Ventilator; Harvard Apparatus, United States) during surgeries. After left lateral thoracotomy, the left anterior descending coronary artery (LAD) was determined by the cross line of venae cordis magna brunches and the left auricle, LAD was occluded in the same point permanently for ischemia with an 8-0 nylon suture and polyethylene tubing was used for preventing arterial injury. Depending on different groups, mice were injected with MFA (10 mg/kg) or vehicle (DMSO) for 28 days after surgery and observed the death rate in each group meanwhile. At the end of experiments, six mice in each group were chosen randomly for next experiments. Mice hearts were excised on condition of anesthetizing with 3% isoflurane and ventilating, the ischemic region (fibrosis region) of the left ventricle was separated and stored at −80°C to further immunoblotting analysis or fixed in 4% formaldehyde to further issue staining or immunofluorescence. All the procedures were performed in compliance with the Guide for the Care and Use of Laboratory Animals by the US National Institutes of Health (NIH Publication, revised in 2011) and approved by the Animal Care and Use Committee of Renji Hospital of Shanghai Jiao Tong University.

### Histological Analysis

Tissue staining and immunohistochemistry were performed to observe the morphological changes of left ventricular (LV), ischemic region (fibrosis region) under the nylon suture knot was separated and fixed in 4% formaldehyde immediately. After embedding and slicing in paraffin, hematoxylin-eosin (HE) staining was performed to observe the tissue morphologies of fibrosis region. Masson and Sirius red staining were performed to determine and compare the differences of collagen deposition among tissues. To perform immunohistochemistry, xylene was used for eluting paraffin. Anti-E2F1 (1:200), anti-CCNE2 (1:200), anti-RhoA (1:50), and anti-ROCK2 (1:200) antibodies were incubated for observing and comparing the differences of the expression of proteins mentioned above in normal region and fibrosis region. Images were captured by an inverted light microscope (magnification: ×100, Olympus, Japan).

### Immunofluorescence

LVs were separated from mouse heart and embodied by paraffin; after slicing and pasting to glass side, embodied sections of LVs were eluted by xylene; after antigen repairing in Citrate Antigen Retrieval solution (Thermofisher, United States), sections were penetrated by 0.3% PBST for 30 min; after washing three times in 0.1% TBST for 15 min, anti–α-SMA (Mouse, 1:200) and anti-FN-1 (Rabbit, 1:200) antibodies were mixed and incubated under condition of 4°C overnight, and then the sections were mixed and incubated in different spices of Alexa-fluor–labeled secondary antibodies (Goat to mouse and goat to rabbit) for 30 min away from light after washing three times in PBST for 30 min. For observing and comparing the differences of the expression of proteins mentioned above in the normal region and the fibrosis region, a fluorescence microscope (magnification: ×400; Olympus, Japan) was used for capturing the fluorescence images.

### Echocardiography

Mice were anesthetized at rest with 3% isoflurane before all hairs in chest were removed. A high-resolution imaging system for small animals (Vevo 3100 imaging system, Visual Sonics Japan) was used in both the B-mode and the M-mode for evaluating cardiac function of mice and capturing echocardiographic images. Parasternal long-axis and short-axis views were both captured. The cardiac function data were calculated by Vevo 3100 software (Visual Sonics Japan).

### Data Analysis and Statistics

Data in bar charts were showed as mean ± SD. For continuous variables (like the gray level of Wb gels, Cp value of qPCR, and a measurement value of cardiac function), a non-paired student’s t-test was used for comparing two different groups and one-way-ANOVA was used for comparing multiple (>2) groups. For discontinuous variables (like cell count and cell percentage), a chi-square test was used for comparing different groups. P value < 0.05 was considered as statistically significant difference and all analyses were performed with GraphPad Prism 7.

## Results

### MFA-Attenuated Cell Differentiation in TGF-β1–Stimulated HCFs

**FIGURE 1:**
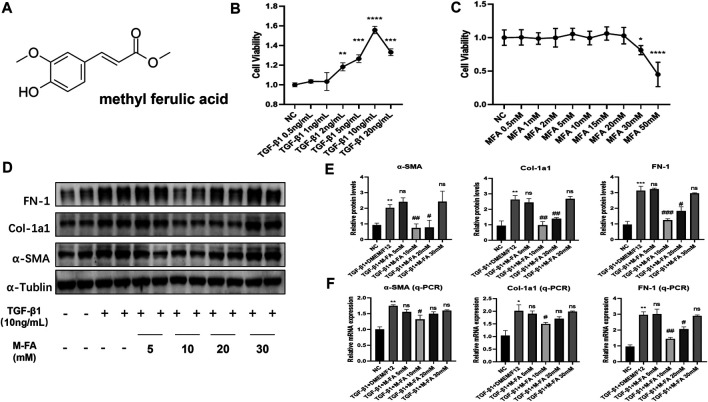
MFA reduced fibrosis-related genes expression in TGF-β1–stimulated HCFs. **(A)** Chemical structural formula of MFA. **(B**–**C)** Cell viabilities when HCFs were treated by different concentrations of TGF-β1 (B) and MFA (C) (*n* = 6). **(D**–**F)** Protein (D. E) and mRNA (F) expression levels of fibrosis-related genes (*n* = 4). (*compared with negative control: *p < 0.05, **p < 0.01, ***p < 0.005, ****p < 0.001. ^#^Compared with the TGF-β1 group: ^#^ *p* < 0.05, ^##^ *p* < 0.01, ^###^ *p* < 0.005).

MFA was an organic acid extracted from traditional Chinese herbs (Figure 1A). To elucidate the effect of MFA in HCF differentiation, TGF-β1 was used for inducing the differentiation of HCFs guided by our formal experiences (Cui et al., 2020), 6-level concentrations of TGF-β1 were incubated with HCFs. After 24 h of incubation, the CCK-8 assay was performed to determine the cell viability in different TGF-β1 concentrations. Our data showed that 10 ng/ml was the most effective concentrations of TGF-β1 in stimulating HCFs (Figure 1B). To test the cytotoxicity of MFA, the CCK-8 assay was also performed to determine the cell viability in nine levels of MFA concentration. Our data showed that the concentrations of MFA were noncytotoxic until 30 mM (Figure 1C). To elucidate the effect of MFA in TGF-β1–stimulated HCFs, 10 ng/ml of TGF-β1 was added, respectively, into 5, 10, 20, and 30 mM concentrations of MFA-treated HCFs. The Western blot analysis and qPCR were both performed in protein and mRNA levels to evaluate the expression of differentiation-related proteins. FN-1, Col-1a1, and α-SMA were chosen to reflect the ECM secretion and the differentiation level. Our data showed that after pretreating with four different levels of MFA, 10 mM of that was the most effective concentrations in reducing differentiation-related proteins expression on TGF-β1-stimulated HCFs (Figures 1D–F), while MFA treated only did not exert any impact on differentiation of HCFs. Our findings showed that MFA reduced differentiation-related proteins expression in TGF-β1–stimulated HCFs and hence suppressed the differentiation of HCFs.

### MFA Suppressed Proliferation and Migration Ability in TGF-β1–Stimulated HCFs

**FIGURE 2:**
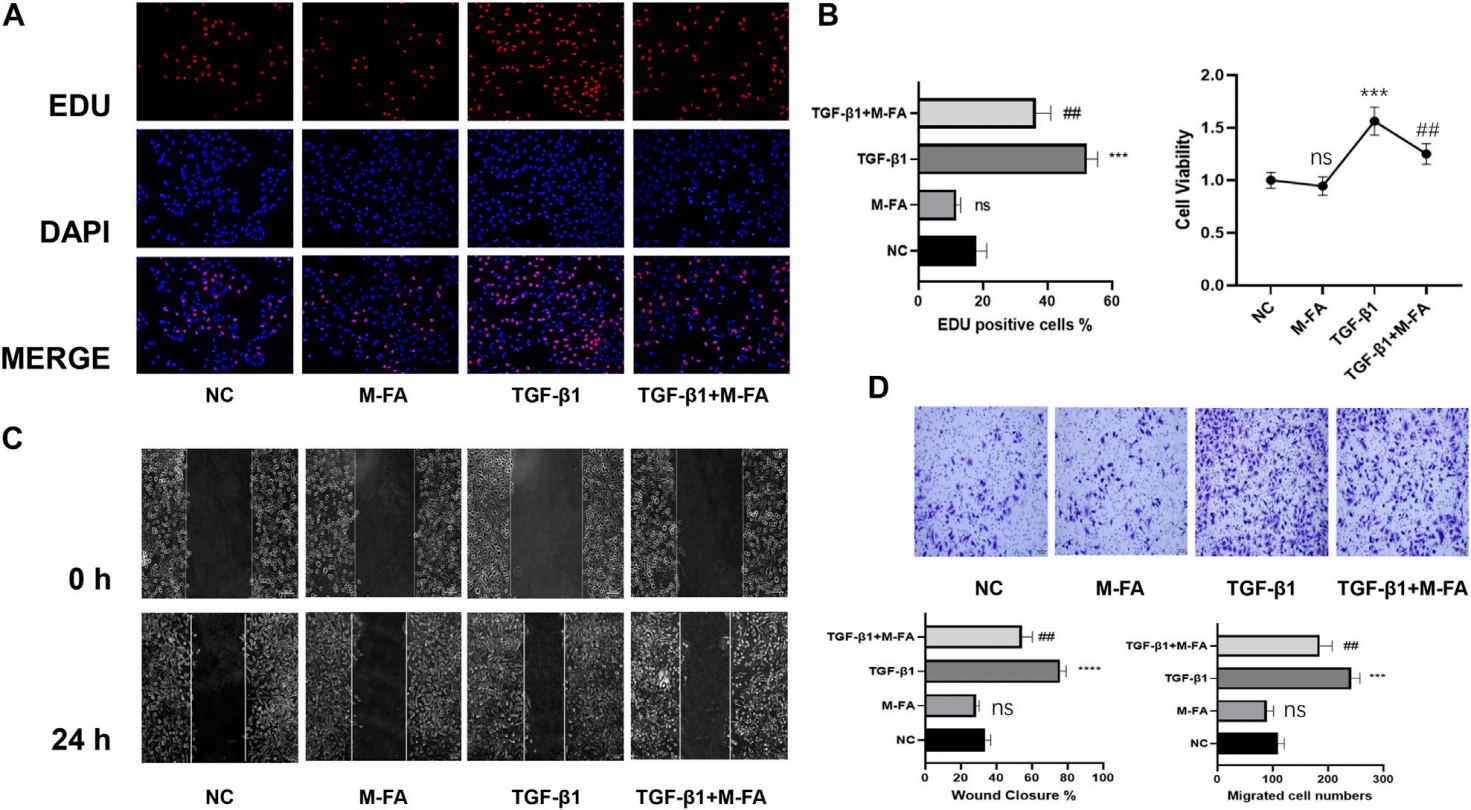
MFA suppressed proliferation and migration ability in TGF-β1–stimulated HCFs. **(A**–**B)** EDU assay (A) and CCK-8 assay (B) were performed to evaluate the proliferation ability of HCFs (*n* = 6). **(C**–**D)** Wound healing assay (C) and Transwell assay (D) were performed to evaluate the migration ability of HCFs treated as below (*n* = 6). (*compared with negative control: ***p < 0.005, ****p < 0.001. ^#^Compared with the TGF-β1 group: ^#^ *p* < 0.05, ^##^ *p* < 0.01).

Both proliferation and migration ability played key roles in differentiation of HCFs. To further elucidate the effect of MFA in HCFs, proliferation and migration ability of HCFs were also measured. By means of the CCK-8 assay and the EDU assay, our data showed that when HCFs were incubated with 10 ng/ml of TGF-β1 and 10 mM of MFA, the cell viability and proliferation ability were both suppressed compared to those who incubated with TGF-β1 only (Figures 2A,B), which meant that MFA suppressed proliferation ability of TGF-β1–stimulated HCFs. Similarly, by means of a wound healing assay and the transwell assay, our data showed that the cell migration ability were both suppressed when HCFs were incubated with TGF-β1 and MFA compared to those who incubated with TGF-β1 only (Figures 2C,D), while MFA treated only did not influence proliferation or migration ability either. Proliferation and migration assays were both identified that MFA suppressed proliferation and migration ability of TGF-β1–stimulated HCFs, which further identified that MFA suppressed the differentiation of TGF-β1–stimulated HCFs.

### MFA Reduced Fibrosis Level in MI Mice

**FIGURE 3:**
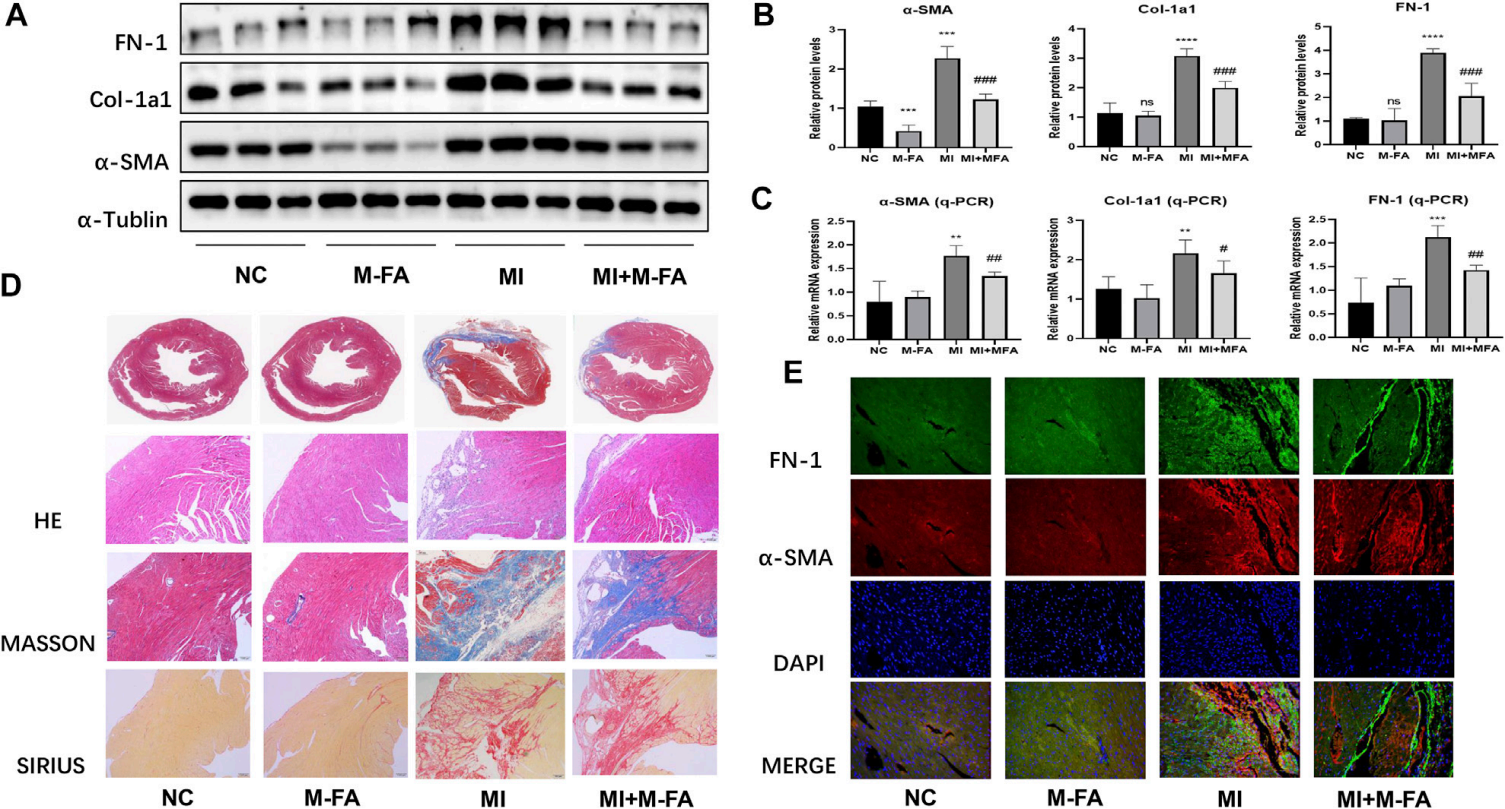
MFA reduced fibrosis level in MI mice. **(A**–**C)** Protein (A. B) and mRNA (C) expression levels of fibrosis-related genes in different mice groups (*n* = 6). **(D**–**E)** Tissue staining (D), and immunofluorescence (E) were performed to compare the fibrosis area and fibrosis-related genes expression. (*compared with negative control: **p < 0.01, ***p < 0.005, ****p < 0.001. ^#^Compared with the MI group: ^#^ *p* < 0.05, ^##^ *p* < 0.01, ^###^ *p* < 0.005).

To elucidate the effect of MFA *in vivo*, MI mice were built to observe the effect of MFA in improving fibrosis-related genes expression and fibrosis areas. To elucidate the effect of MFA in improving fibrosis-related genes expression, MI mice were injected intraperitoneally with 10 mg/kg concentrations of MFA solutions per day. After 28 days of injecting, LVs of mice were separated for Western blot and qPCR. Our data showed that all MI mice were successfully modeled, and fibrosis-related genes expression of the MFA-treated group was obviously reduced in both protein and mRNA levels in contrast to the MI group (Figures 3A–C). Furthermore, to evaluate the effect of MFA in improving fibrosis areas, the histological analysis and immunofluorescence were performed to observe the differences of fibrosis areas. By means of HE, Masson, and Sirius staining, our data showed that fibrosis areas (bules in Masson and reds in Sirius) were all decreased in the MFA-treated group in contrast to the MI group (Figure 3D) and to determine the constituents in the fibrosis area, immunofluorescence was performed to show the expression of FN-1 and α-SMA in fibrosis. Our data showed that in fibrosis areas, both FN-1 and α-SMA were upregulated, but the expression level and the fibrosis area were reduced in the MFA-treated group in contrast to the MI group (Figure 3E; Supplementary Figure 2A). Certainly, MFA-treated mice without MI were not showed obvious differences to the NC group in fibrosis-related genes expression excepted α-SMA. Our findings showed that MFA reduced the fibrosis-related genes expression and fibrosis areas in MI mice, meant that MFA reduced fibrosis levels *in vivo*.

### MFA Improved the Survival and Cardiac Function in MI Mice

**FIGURE 4:**
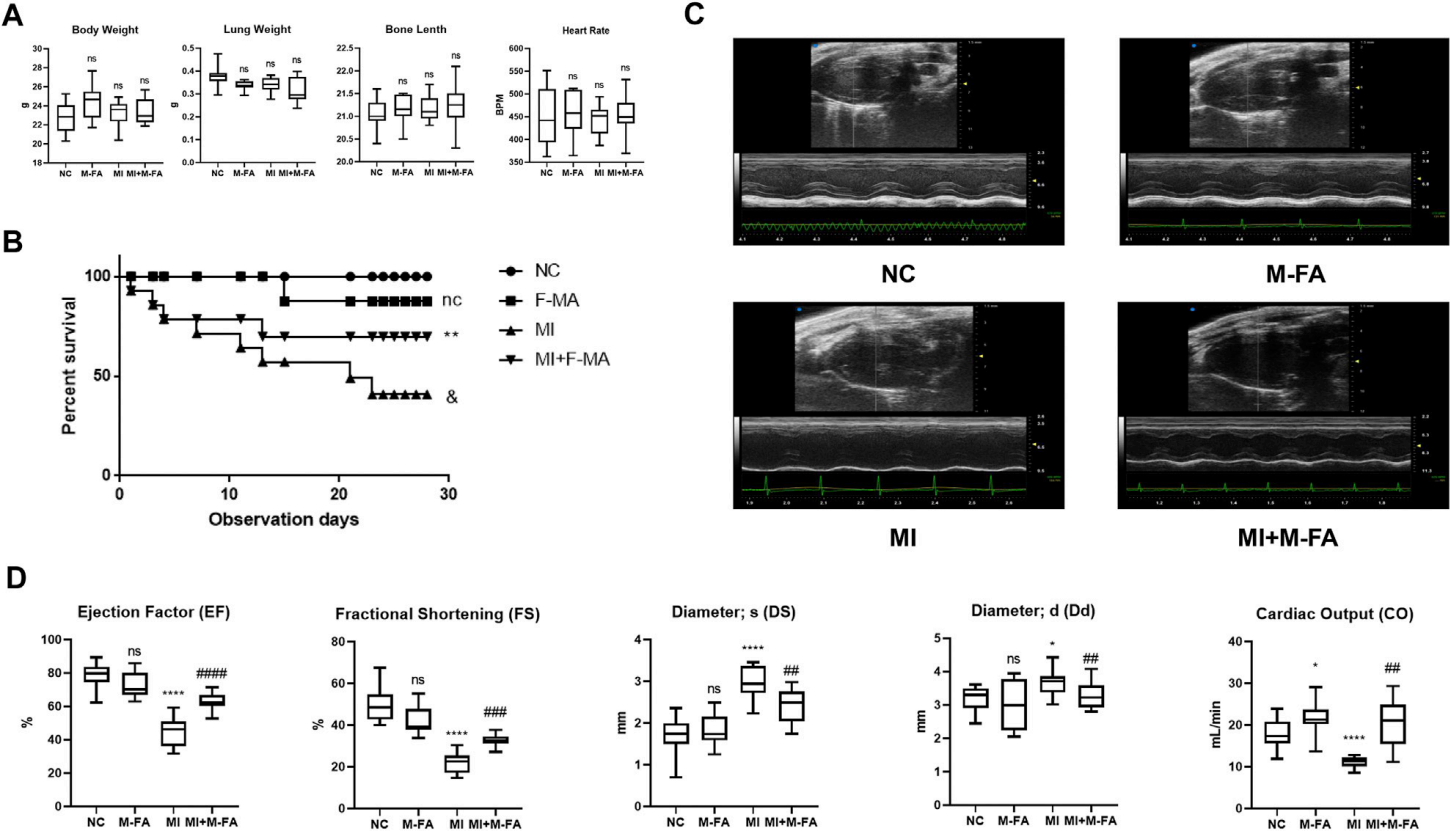
MFA improved the survival and cardiac function in MI mice. **(A)** General indicator among four groups of mice (*n* = 6). **(B)** K-M analysis among four groups of mice (n = 15). **(C**–**D)** Echocardiography images (in both B and M mode) (C), and cardiac functions among four groups of mice (*n* = 6). (*compared with negative control: *p < 0.05, **p < 0.01, ****p < 0.001. ^#^Compared with the MI group: ^#^ *p* < 0.05, ^##^ *p* < 0.01, ^###^ *p* < 0.005, ^####^ *p* < 0.005).

When MI occurred, heart chamber architecture changed significantly due to myocardial fibrosis, hence led to heart failure at the terminal stage. To further evaluate the effect of MFA in reducing fibrosis and protecting myocardium, echocardiography was performed to evaluate the cardiac function of mice. Before evaluating, the body weight, the lung weight, the bone length, and the heart rate were controlled without significant differences among four groups to exclude the influences from them on results (Figure 4A). Firstly, by a survival analysis, we found that survival in 28 days was significant decreased in mice after MI, but improved after MFA treatment, especially at the terminal stage (Figure 4B). By analyzing the outcomes of echocardiography, we surprisingly found that the main cardiac function indexes (the ejection factor and fractional shortening) were significantly improved in the MFA-treated group in contrast to the MI group**.** Besides, some secondary cardiac function indexes (Ds, Dd, and CO) were also improved (Figures 4C,D). MFA-treated mice without MI were not showed the obviously differences to the NC group in these indexes. Our findings showed that MFA improved the survival and cardiac function in MI mice. Combined with our formal findings, we determined that MFA improved the outcomes of fibrosis after MI *in vitro*.

### MFA Suppressed the Expression of pRB, E2F1, CCNE2, RhoA, and ROCK2, Regulated the Cell Cycle, and Conjunction of RB and E2F1

**FIGURE 5:**
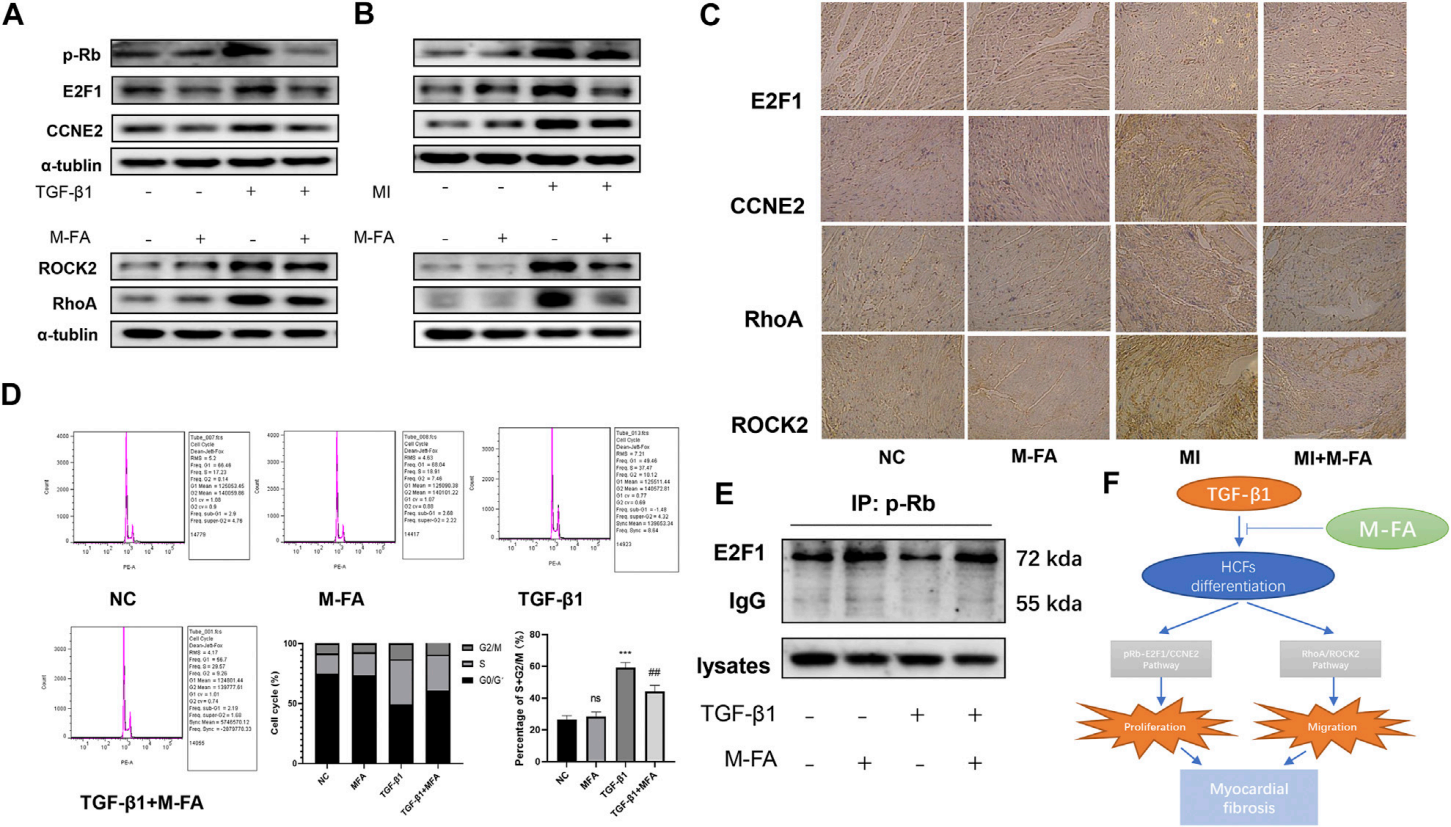
MFA suppressed the expression of pRB, E2F1, CCNE2, RhoA, and ROCK2, regulated the cell cycle, and conjunction of RB and E2F1. **(A**–**C)** Protein expression levels of proliferation and migration-related genes in HCFs (A), mice LVs (B), and mice tissue (C) (*n* = 3). **(D)** Cell cycle analysis in HCFs (*n* = 6). **(E)** Immunoprecipitation of anti-pRB to E2F1 in HCFs lysates. **(F)** Sketch map of our assumptions. (*compared with negative control: ***p < 0.005. ^#^Compared with the TGF-β1+MFA group: ^##^ *p* < 0.01).

In our previous results, we determined that in HCFs, MFA suppressed its proliferation and migration abilities, as classic cellular signal pathway components, pRB, E2F1, and CCNE2 took parts in cell proliferation and RhoA and ROCK2 took parts in cell migration were widely reported. In this context, we determined to assess the expression of pRB, E2F1, CCNE2, RhoA, and ROCK2. In TGF-β1–stimulated HCFs, we found that TGF-β1 promoted the expression of pRB, E2F1, CCNE2, RhoA, and ROCK2, but this effect could be attenuated by MFA pretreatment (Figure 5A; Supplementary Figure 2B). The same phenomenon was also observed in left ventricular lysates of MI mice (Figure 5B; Supplementary Figure 2C). Then, immunohistochemistry was performed to observe the expression of those components in mice hearts. By means of microscopy we found that E2F1, CCNE2, RhoA, and ROCK2 were all high expressed in fibrosis areas of the MI group but attenuated in fibrosis areas of the MFA-treated group (Figure 5C). pRB, E2F1, and CCNE2 were key components of cell cycle, hence the cell cycle analysis (by flow cytometry) was performed to further elucidated the relationship between MFA and the cell cycle. Our data showed that TGF-β1 promoted the cell proliferation by improving the percentage of S and G2/M phase cells, the effect could also be attenuated by MFA pretreatment (Figure 5D). Besides, RB was conjunct with E2F1 in common circumstance but phosphorylated (pRB) and separated from E2F1 when stimulated by TGF-β1. This phenomenon was observed by immunoprecipitation. However, we found that the separation was also attenuated by MFA pretreatment (Figure 5E). These findings showed that MFA suppressed the expression of pRB, E2F1, CCNE2, RhoA, and ROCK2, regulated the cell cycle, and conjunction of RB and E2F1, not surprisingly, MFA-pretreated HCFs and mice without TGF-β1 stimulating or MI modeling were not showed those changes. Following these findings, we assumed that MFA attenuated proliferation and migration ability of HCFs and thus attenuated myocardial fibrosis by suppressing the pRB-E2F1/CCNE2 and the RhoA/ROCK2 pathway (Figure 5F).

### MFA Suppressed Proliferation and Migration Ability of HCFs and Attenuated Myocardial Fibrosis by Suppressing the RB-E2F1/CCNE2 and the RhoA/ROCK2 Pathway

**FIGURE 6:**
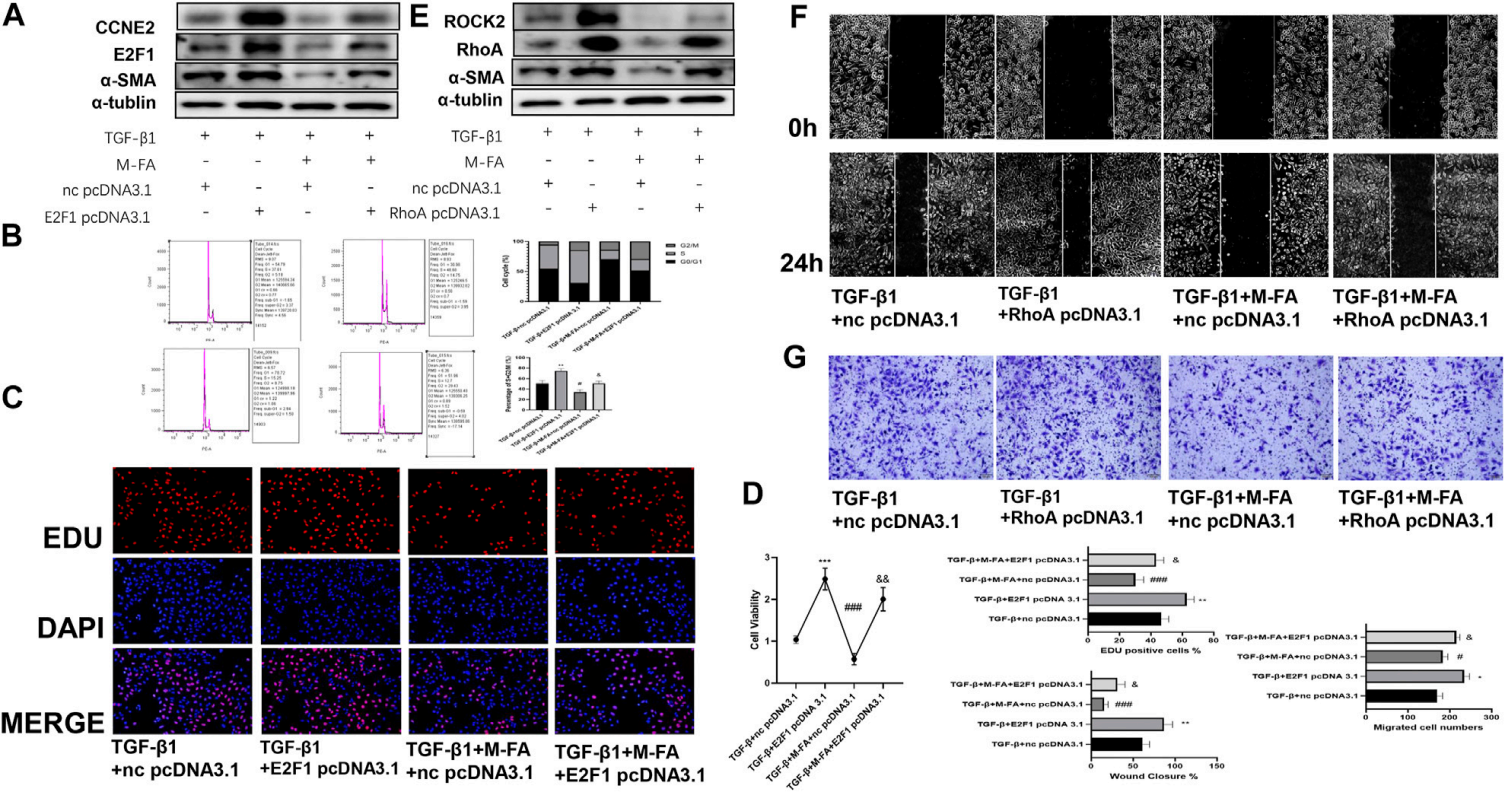
MFA suppressed the expression of pRB, E2F1, CCNE2, RhoA, and ROCK2, regulated the cell cycle, and conjunction of RB and E2F1. **(A**–**D)** Rescue test for cell proliferation ability and ECM formation by Western blot (A) (*n* = 3), cell cycle analysis (B), EDU assay (C), and CCK-8 assay (D) (*n* = 6). **(E**–**G)** Rescue test for cell migration ability and ECM formation by Western blot (E) (*n* = 3), Wound healing assay (F), and TRANSWELL assay (*n* = 6) (G). (*compared with negative control: *p < 0.05, **p < 0.01, ***p < 0.005. ^#^Compared with the TGF-β1 group: ^#^ *p* < 0.05, ^###^ *p* < 0.005. ^&^Compared with the TGF-β1 + MFA group: ^&^ *p* < 0.05, ^&&^ *p* < 0.01).

To further elucidated the relationships between suppressed proliferation and migration ability of HCFs by MFA with the RB-E2F1/CCNE2 and the RhoA/ROCK2 pathway, pcDNA 3.1 of E2F1, and RhoA were designed to rescue the suppressed pathway components. Firstly, E2F1 pcDNA 3.1 was transfected transiently into TGF-β1 and MFA-treated HCFs, and the ECM formatting (evaluated by α-SMA expression), cell proliferation (evaluated by EDU assay and CCK-8 assay) ability, and the cell cycle were all recovered. Besides, CCNE2, the downstream molecule of E2F1 was also recovered (Figures 6A–D; Supplementary Figure 2D). Same procedure was performed by RhoA pcDNA 3.1, after transfecting, cell migration ability (evaluated by the wound healing assay and the transwell assay) and downstream molecule ROCK2 were also recovered (Figures 6E–G; Supplementary Figure 2E). Furthermore, to ensure the activity of ROCK2, we detected the expression of phosphorylated MYPT1 (p-MYPT1), the substrate of ROCK2. We found that p-MYPT1 was highly expressed in HCFs stimulated by TGF-β1, but attenuated with MFA pretreatment, which consisted with that of ROCK2 (Supplementary Figures 1A, 2F). Rescue tests by RhoA pcDNA 3.1 showed that with recovering of ROCK2 expression, p-MYPT1 expression was also recovered (Supplementary Figures 1B, 2G), meant that the RhoA/ROCK2 pathway was activated in TGF-β1–stimulated HCFs and deactivated when MFA treatment. At last, to further proved the roles of the RB-E2F1/CCNE2 and the RhoA/ROCK2 pathway in TGF-β1–induced cell differentiation, the inhibitors of the RB-E2F1/CCNE2 pathway (MRT00033659) and the RhoA/ROCK2 pathway (Fasudil) were purchased to conduct the experiment, results showed that after treating by inhibitors, cell proliferation (evaluated by EDU assay), and migration (evaluated by Transwell assay) ability were all attenuated in contrast to the TGF-β1–stimulated group (Supplementary Figures 1C,D), meant that those two pathways were participated in TGF-β1–stimulated cell differentiation. With these findings, we concluded that MFA suppressed proliferation and migration ability of HCFs and hence attenuated myocardial fibrosis, the mechanism of that was by suppressing the RB-E2F1/CCNE2 and the RhoA/ROCK2 pathway.

## Discussion

When myocardial fibrosis occurred, intense inflammatory responses would happen due to myocardial necrotizing, and the responses initiated the process of scar repairing in infarcted heart areas, scar repairing response was essential for maintaining the structural and functional integrity of heart tissue, but excessive deposition of ECM caused by fibroblasts-to-myofibroblasts shifts worsen the outcomes of myocardial infarction and led to myocardial dysfunction and heart failure at the terminal stage (Pchejetski et al., 2012; Gao et al., 2019; Rodriguez et al., 2019). TGF-β1 induced differentiation of HCFs was regarded as a critical factor of this process (Zeng et al., 2019; Parichatikanond et al., 2020). When TGF-β1 was activated, it bounded with the receptor TβRI and TβRII, TβRI bounded with its downstream transcription factor SMAD4, and TβRII phosphorylated SMAD2/3, which combined with SMAD4 and entered into cell nucleus to promote transcription of fibrosis–related genes (Khalil et al., 2017). In our research, we found that after stimulated by TGF-β1, cell viability, fibrosis-related genes expression, cell proliferation, and migration abilities were all enhanced, which was in line with reports and our previous publication (Cui et al., 2020).

Massive endeavors were made to relive or reverse myocardial fibrosis (Yang et al., 1999; Cunnington et al., 2009; Lei et al., 2011; Khalil et al., 2017), but the effect of those treatment strategies was failed because targeting TGF-β1, SMAD2/3, or TβRI/II directly will be ended up with poor outcomes like autoimmune diseases, heart failure, or unknown risks (Shull et al., 1992; Wang et al., 2005). Another treatment strategy should be considered to tackle this problem. Some research groups reported that non-coding RNAs might had effects on reliving myocardial fibrosis both *in vivo* and *in vitro* (Bejerano et al., 2018; Seo et al., 2019; Zhang et al., 2021), but non-coding RNA treatments were relatively high cost, and the effects were unclear. What’s more, non-coding RNA treatments might exert several potential application problems and side effects, like the innate immune system activating and severe toxicity (Poller et al., 2013); relatively safe and accessible strategies were needed to be further developed. As an organic acid extracted from traditional Chinese herbs, MFA was reported by some research groups for its roles in organ protection and anti-inflammation (Li et al., 2017; Li et al., 2018; Cheng et al., 2019a; Cheng et al., 2019b). In our results, we found that low concentration of MFA was relatively safe to HCFs and mice, but most importantly, MFA attenuated the differentiation of HCFs in both fibrosis related genes change and cell behavior change (decreased cell proliferation and migration abilities) *in vivo*. Besides, MFA improved myocardial fibrosis and cardiac function *in vitro*. Our findings were in line with previous reports.

E2F1 was a key member of E2F family, those transcription factors played important roles in many cell activities like DNA damage repair, cell apoptosis, and cell cycle progression. The members of E2F family exerted both gene activation and repression effect. E2F1-3a mainly played the roles of gene activation while E2F3a, E2F4-8 played the opposite effect (Trimarchi and Lees, 2002; Kent et al., 2016; Xiang et al., 2019). As the first elucidated and the most known members of E2F family, E2F1 was tightly associated with cell death, cell proliferation, and liver fibrosis. E2F1 worked as complexes with RB, when activated; RB was phosphorylated and separated from E2F1, and E2F1 got into cell nucleus to transcript downstream genes (Zhang et al., 2014; Lai et al., 2017). As a downstream transcriptional target, CCNE2 was also involved in cell cycle and cell proliferation. CCNE2 was a member of Cyclin E family and CCNE2 with the other Cyclin E family members functioned alike, which activated the CDK2 to promote cell cycle progression (Geng et al., 1996; Lee et al., 2020). In our results, we found that in TGF-β1–stimulated HCFs, the cell proliferation ability was enhanced and the cell cycle was promoted by improving percentages of the S and G2/M phase. Consequently, the expression of p-RB, E2F1, and CCNE2 were synchronous increased, by means of IP, the conjunction amount of p-RB and E2F1 were reduced compared to the negative control. The phenomenon was inverted when pretreated by MFA. By means of a rescue test, we further determined that MFA suppressed the TGF-β1–stimulated cell proliferation ability enhanced by suppressing the pRB-E2F1/CCNE2 pathway. The RhoA/ROCK pathway is composed by RhoA, the most representative subtype of small G proteins of Rho family, and its downstream molecule ROCK1/2. Rho family was widely involved in regulating cell functions like migration, adhesion, cell survival, and apoptosis (Amano et al., 2010). As a subtype of ROCK, ROCK2 was expressed in many tissues excepted nervous tissue and smooth muscle (Zhou and Liao, 2009). In our results, we found that in TGF-β1–stimulated HCFs, cell migration ability was enhanced, expression of RhoA and ROCK2 was also increased, and the phenomenon was also inverted by MFA, but recovered in the rescue test. All our results above were proved our hypotheses before.

Finally, our findings strongly identified that by suppressing the pRB-E2F1/CCNE2 and the RhoA/ROCK2 pathway, MFA attenuated the ability of migration and proliferation, attenuated the expression of fibrosis-related proteins in HCFs, and improved the cardiac function in MI mice meanwhile. But interestingly, we found that when mice injected by MFA without MI, the expression of α-SMA was significantly decreased, from other reports we knew that ferulic acid (FA), the ramification of MFA, suppressed the expression of fibroblast growth factor receptor 1 (FGFR1), and hence suppressed the angiogenesis and α-SMA expression (Yang et al., 2015; Chen et al., 2019). Although the effect of MFA on FGFR1 was not reported, we assumed that it might play the same role as FA. Certainly, the mechanism of how MFA suppressed the expression of those genes needed to be further elucidated.

## Data Availability

The original contributions presented in the study are included in the article/Supplementary Material; further inquiries can be directed to the corresponding authors.

## Supplementary Material

Supplementary Figure 1 **(A)** Protein expression levels of ROCK2 and p-MYPT1 (*n* = 3). **(B)** Rescue test for determining the activity of RhoA and ROCK2 (*n*=3), **(C, D)** EDU assay **(C)** and Transwell assay **(D)** for determining cell proliferation and migration abilities (*n* = 6). (*compared with negative control: **p* < 0.05, ***p* < 0.01, ****p* < 0.005. ^#^Compared with the TGF-β1 group: ^#^ *p* < 0.05, ^###^ *p* < 0.005. ^&^Compared with the TGF-β1 + MFA group: ^&^ *p* < 0.05, ^&&^ *p* < 0.01)

Supplementary Figure 2 **(A)** Relative fluorescence intensities of α-SMA and FN-1 in MI mice (*n* = 6). **(B, C)** Protein expression levels of proliferation and migration related genes in HCFs **(B)** and mice LVs **(C)** (*n* = 3). **(D, E)** Rescue test for cell proliferation **(D)** and migration **(E)** abilities and ECM formation by Western blot **(A)** (*n* = 3). **(F)** Protein expression levels of ROCK2 and p-MYPT1 (*n* = 3). **(G)** Rescue test for determining the activity of RhoA and ROCK2 (*n* = 3). (*compared with negative control: **p* < 0.05, ***p* < 0.01, ****p* < 0.005, *****p* < 0.001. ^#^Compared with the TGF-β1 group: ^#^ *p* < 0.05, ^##^ *p* < 0.01, ^###^ *p* < 0.005, ^####^ *p* < 0.001. ^&^Compared with the TGF-β1+MFA group: ^&^ *p* < 0.05, ^&&^ *p* < 0.01, ^&&&^ *p* < 0.005).
