# Supplementary figures and images for: Methyl Ferulic Acid Attenuates Human Cardiac Fibroblasts Differentiation and Myocardial Fibrosis by Suppressing pRB-E2F1/CCNE2 and RhoA/ROCK2 Pathway
Source: Front Pharmacol. 2021 Aug 17;12:714390. doi: 10.3389/fphar.2021.714390 (PMC8416034; doi:10.3389/fphar.2021.714390)

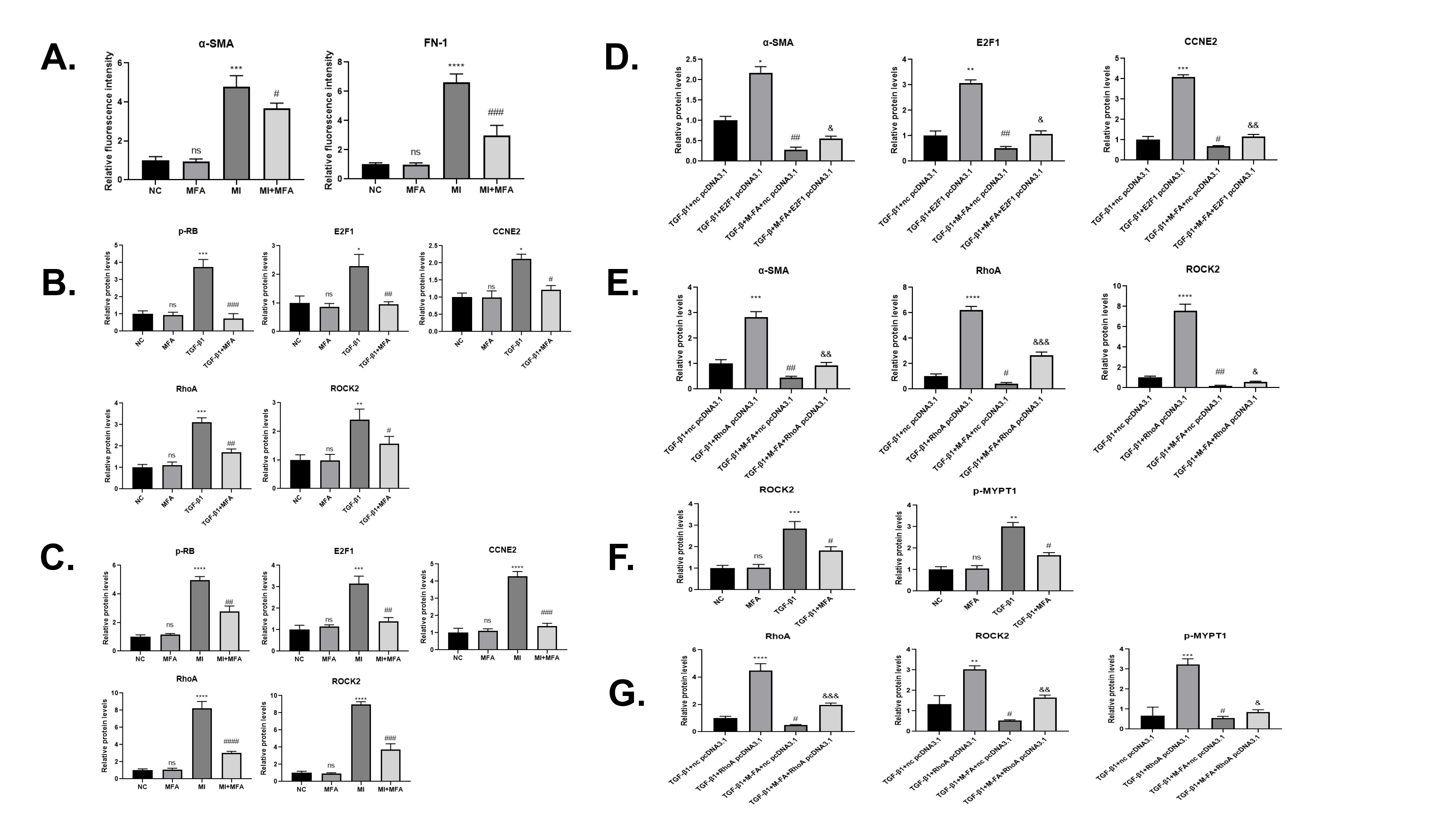

Supplement: Supplementary file 1 [file Image2.TIF]

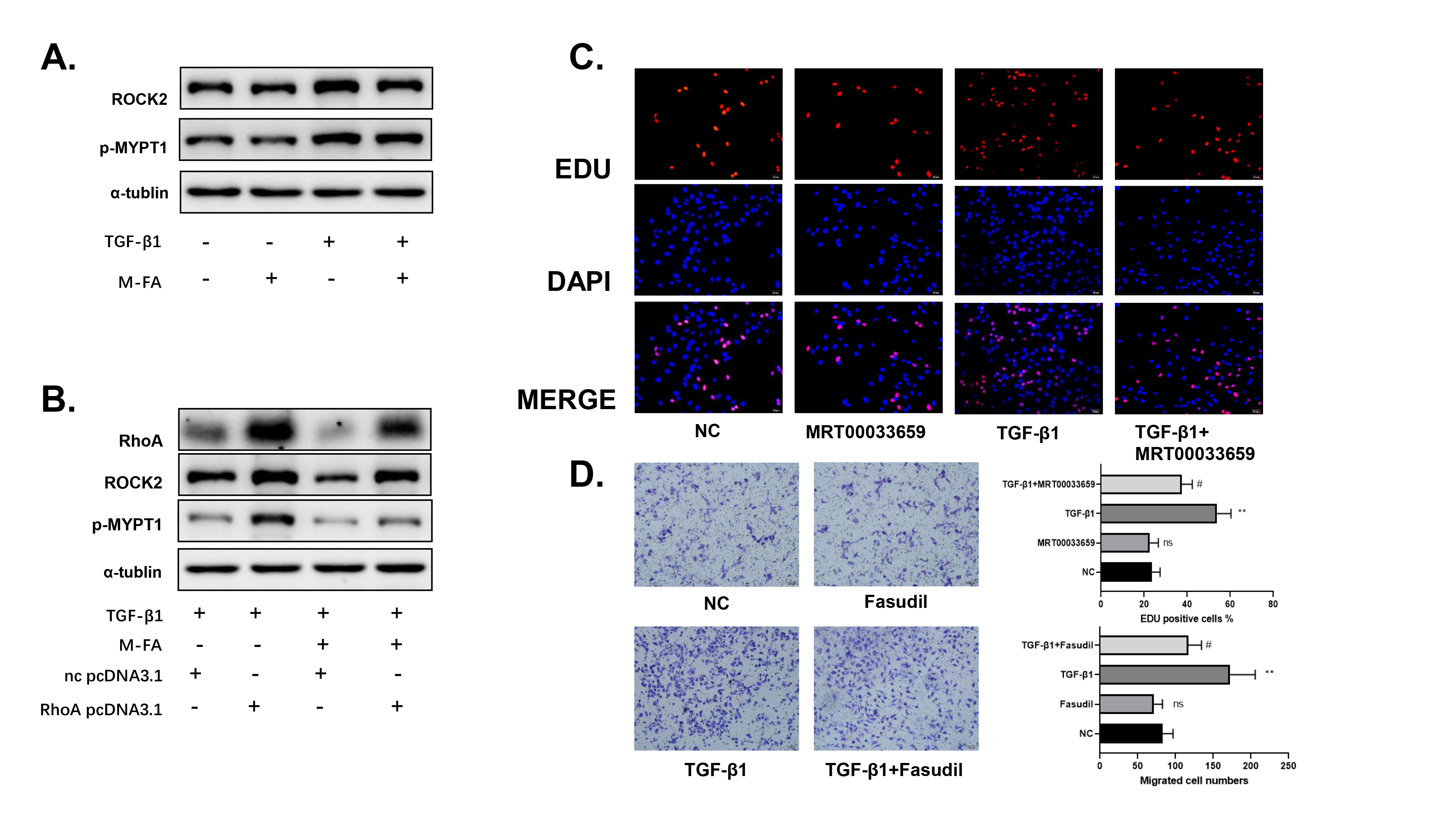

Supplement: Supplementary file 2 [file Image1.TIF]
