# Supplementary material for: Methyl Ferulic Acid Attenuates Human Cardiac Fibroblasts Differentiation and Myocardial Fibrosis by Suppressing pRB-E2F1/CCNE2 and RhoA/ROCK2 Pathway
Source: Front Pharmacol. 2021 Aug 17;12:714390. doi: 10.3389/fphar.2021.714390 (PMC8416034; doi:10.3389/fphar.2021.714390)
Supplement: Supplementary file 3 [file DataSheet1.PDF]

Table 1: sequences of primers

Genes	Sequences
GAPDH	F: CCTCGCTCTAGGGAGGTTTTA<br>R: CCGACAACAGTATGAAGAGTACC
α-SMA	F: TTTTCTGTCGATGCACCCACT<br>R: CGGTACAAGATAGCCCATGAA
COL1A1	F: CTCCCGGTTCTGCTTCTGTAG<br>R: GTCTAGTGCAGTAGCGTGTTG
FN1	F: GCCACCGACAGTCAGTTTC<br>R: TTTGGAGCCGAAGGAGGTATT
pRB	F: CGGCTATCGGTATGCTGATTTA<br>R: CTTAATCGGTGATCGTAGCTAG
E2F1	F: ACGCTATGAGACCTCACTGAA<br>R: TCCTGGGTCAACCCCTCAAG
CCNE2	F: TATTTCGCTACTCCCTTGATCTGA<br>R: GCCGTATACGTGGTCGTAGTCGA
RhoA	F: CGGAGATTTACGTAGCTGTTCAAC<br>R: CGGCTAGCTAGCCTCTGTGATCGT
ROCK2	F: ATTCGTACGTCGACCTGTGAACTGT<br>R: CTGATGCTGTAATTCGGTGATTACC
